# Differences in Rejuvenation Mechanisms and Physical Properties of Aged Styrene–Butadiene–Styrene (SBS)-Modified Bitumen by Mono-Epoxy and Di-Epoxy Compounds

**DOI:** 10.3390/polym17010086

**Published:** 2024-12-31

**Authors:** Kingsley C. K. Chiang, Bohan Zhu, Lingxiao Liu, Haozongyang Li, Cheng Chen, Shixian Tang, Chengwei Xing

**Affiliations:** 1 China State Construction Engineering (Hong Kong) Limited, Hong Kong 999077, China; kingsleychiang@cohl.com (K.C.K.C.); lingxiao.liu@cohl.com (L.L.); chen_cheng@cohl.com (C.C.); 2 Key Laboratory for Special Area Highway Engineering of Ministry of Education, Chang’an University, South 2nd Ring Road Middle Section, Xi’an 710064, China; lihzy@chd.edu.cn (H.L.); xingcw@chd.edu.cn (C.X.); 3 School of Highway, Chang’an University, South 2nd Ring Road Middle Section, Xi’an 710064, China

**Keywords:** SBS-modified bitumen, rejuvenator, reactive compound, rheological property

## Abstract

Studying the mechanisms and effects of rejuvenators on SBS-modified bitumen is crucial for repairing degraded SBS and recycling aged SBS-modified bitumen (ASMB), thereby contributing to the sustainable development of bitumen pavements. This research examines the roles of mono-epoxy Alkyl (C12-C14) glycidyl ether (AGE) and di-epoxy 1,6-Hexanediol diglycidyl ether (HDE) under the catalysis of N,N-dimethyl benzyl amine (BDMA) in repairing degraded SBS chains. Aromatic oil (ORSMB)-, AGE–aromatic oil (ARSMB)-, and HDE–aromatic oil (HRSMB)-rejuvenated bitumen are analyzed for their chemical structures, physical properties, and rheological properties. Fluorescence microscopy (FM) and Fourier transform infrared spectroscopy (FTIR) reveal that HDE chemically reconnects degraded SBS chains, enhancing ASMB properties, while AGE improves ASMB properties through physical softening. HDE balances high-temperature properties and improves mid-temperature fatigue resistance through a rigid repair effect and flexible chain structure. AGE enhances mid-temperature fatigue resistance but significantly reduces high-temperature rutting resistance due to a softening effect. The findings demonstrate that HDE restores ASMB ductility chemically, while AGE improves crack resistance through physical softening. These differences in rejuvenation mechanisms provide a theoretical basis for optimizing rejuvenator design and advancing bitumen pavement recycling.

## 1. Introduction

Styrene–butadiene–styrene (SBS) is a styrene–butadiene–styrene block copolymer, also known as a thermoplastic elastomer. SBS is widely used as a bitumen modifier in road construction and rehabilitation and is known for enhancing flowability, crack resistance, and durability under both high- and low-temperature conditions [1]. By enhancing the physical properties of bitumen, SBS significantly prolongs pavement service life and improves fatigue resistance, making SBS a vital component in modern transportation infrastructure [2,3]. During long-term service, SBS-modified bitumen undergoes aging, which diminishes fatigue resistance, rutting resistance, and low-temperature crack resistance [4,5,6]. This deterioration reduces road durability and directly impacts both service life and traffic safety [7]. The aging process of SBS-modified bitumen involves both the degradation of the SBS and the oxidation of the base bitumen [8]. Since SBS and base bitumen are non-renewable resources, managing large volumes of aged bitumen poses significant environmental challenges. Against this backdrop, rejuvenating ASMB has emerged as a key area of research [9]. Rejuvenation technologies aim to restore the properties of aged bitumen, promoting efficient resource recycling [10,11]. This not only lowers economic costs but also plays a vital role in environmental protection.

Conventional bitumen rejuvenation techniques generally involve adding oil-based substances to aged bitumen to modify the composition and restore flexibility [10,12]. This physical modification approach can achieve partial rejuvenation of aged bitumen [13]. Uchoa et al. [14] investigated the rheological properties of rejuvenated base bitumen by incorporating epoxidized castor oil and acrylated cashew nut shell liquid into aged base bitumen. Their findings indicated that bio-based rejuvenators significantly softened the aged base bitumen and enhanced the bitumen’s anti-aging properties. Similarly, Zhang et al. [15] utilized residual soybean oil at varying dosages to rejuvenate aged 70# base bitumen. The addition of bio-oil enhanced the light fractions in aged base bitumen, lowered viscosity, and effectively restored the bitumen’s properties, making it suitable for reuse. However, this method shows limited effectiveness in rejuvenating SBS-modified bitumen. While it can restore the flexibility of base bitumen, it fails to recover the high elasticity characteristic of SBS-modified bitumen.

The degradation of SBS fundamentally involves the cleavage of double bonds in polybutadiene. Some studies have explored the addition of light oil fractions and SBS to ASMB. This approach primarily softens aged base bitumen through the light oil fractions while compensating for the loss of SBS in aged bitumen [16]. Although this method effectively improves the high- and low-temperature rheological properties and durability of aged bitumen, it is costly, and the degraded SBS remains unused, leading to resource waste.

In recent years, the development of SBS repair agents has become a research hotspot [17,18,19]. Compounds designed to restore degraded SBS structures are referred to as “reactive compounds”, which are often combined with light oil fractions to rejuvenate ASMB. These combinations are termed “reactive rejuvenators” [20]. Among these, epoxy-based reactive rejuvenators are representative repair agents, named for their epoxy functional groups within the reactive compounds’ molecular structure. Under the action of a catalyst, the epoxy groups undergo ring-opening reactions, chemically bonding with hydroxyl or carboxyl groups at the ends of degraded SBS modifiers, thereby reconnecting broken molecular chains [21,22]. Xu et al. [21] and Yang et al. [19] employed epoxy compounds, including 1,4-butanediol diglycidyl ether (BUDGE) and epoxidized polybutadiene resin (EPR), respectively, to rejuvenate ASMB under catalytic conditions. Their results demonstrated that both epoxy-based reactive compounds effectively repaired degraded SBS and improved the low-temperature properties of aged bitumen. Furthermore, Xu et al. [23] investigated the rejuvenation of ASMB using BUDGE and trimethylolpropane triglycidyl ether (TMPGE), which feature di-epoxy and tri-epoxy structures, respectively. The study revealed that both compounds could restore the original SBS structure, with BUDGE exhibiting superior properties at low temperatures and providing greater flexibility to ASMB. Thus, di-epoxy rejuvenators were found to outperform tri-epoxy rejuvenators in rejuvenation effectiveness.

Epoxy-based reactive compounds have been shown to effectively repair degraded SBS and enhance the low-temperature properties of ASMB. However, the effectiveness of mono-epoxy and di-epoxy structures in repairing degraded SBS, as well as the differences in their rejuvenation effects, remain to be further explored. Based on this, two types of epoxy-based reactive compounds, AGE (mono-epoxy structure) and HDE (di-epoxy structure), were selected in this study. These compounds were combined with aromatic oil and BDMA as a catalyst to rejuvenate ASMB. The aim was to investigate their rejuvenation mechanisms and the property differences in the rejuvenated bitumen. In the experiments, the macro-properties of the rejuvenated bitumen were evaluated using ductility equipment, the Dynamic Shear Rheometer (DSR) test, and the bending beam rheometer (BBR) test, while the micro-mechanism of the rejuvenation process was analyzed using FTIR and FM.

## 2. Materials and Methods

### 2.1. Raw Materials

The base bitumen used in this study was 90# bitumen, shear-mixed with 4.5% by weight of linear SBS. Basic properties of the bitumen are presented in Table 1.

An aromatic oil was used in this study as a rejuvenator for the base bitumen components. Aromatic oil was produced by Beijing Yanxinlian Petrochemical Co., Ltd. The basic properties are shown in Table 2.

The reactive compounds were AGE and HDE, both with a dosage of 1%. The catalyst, BDMA, was added at a dosage of 0.1%. The basic properties of these materials are presented in Table 3.

### 2.2. Preparation of Rejuvenated Bitumen

According to American Society for Testing Materials (ASTM) standards D1754 and D6521 [24,25], the Thin Film Oven Test (TFOT) was used to simulate the short-term aging for 5 h, and a Pressurized Aging Vessel (PAV) was used to simulate the long-term aging for 20 h of bitumen. After aging, the SBS-modified bitumen was heated to 150 °C, and 4% by weight of aromatic oil was added. The mixture was stirred at 150 °C for 15 min at 800 rpm for preliminary blending. Subsequently, 1% by weight of AGE or HDE and 0.1% by weight of BDMA was added, and the mixture was further stirred at 800 rpm for 20 min to ensure the uniform incorporation of the rejuvenator.

### 2.3. Test Methods

#### 2.3.1. Ductility Test

This study conducted the ductility test for the virgin SBS-modified bitumen, ASMB, and rejuvenated bitumen according to the ASTM D113 [26]. All bitumen samples were heated and poured into molds, which were then cooled and removed. The samples were conditioned in a water bath at a constant temperature of 5 °C for 1.5 h. The ductility measurements were conducted using a ductility apparatus. Three parallel tests were performed for each type of bitumen, and the average value was taken as the final result.

#### 2.3.2. Multiple Stress Creep and Recovery Test

The multiple stress creep and recovery (MSCR) test, based on the DSR, is a method for evaluating the high-temperature properties of bitumen, conducted in accordance with AASHTO T350. This study used the MSCR test to evaluate the properties of virgin SBS-modified bitumen, ASMB, and three types of rejuvenated SBS-modified bitumen. The test temperature was controlled within the range of 58–82 °C, increasing in 6 °C increments. Each sample underwent a cycle of 1 s loading followed by 9 s recovery, completing a total of 30 cycles. In the experiment, the first 10 cycles were typically excluded from analysis due to sample adjustment instability, while the data from the final 10 cycles were retained for calculation. Subsequently, the stress was increased to 3.2 kPa to complete the final 10 cycles, providing further evaluation of the material’s resistance to deformation at high temperatures.

#### 2.3.3. Temperature Sweep Test

The temperature sweep (TS) test was conducted to evaluate the rheological properties using a DSR manufactured by TA Instruments, USA. This study used the TS test to evaluate the properties of virgin SBS-modified bitumen, ASMB, and three types of rejuvenated SBS-modified bitumen. The test was performed with a shear strain of 1% and an angular frequency of 10 rad/s. According to ASTM D7175 [27], the test temperature was controlled within the range of 16 °C to 82 °C, with increments of 6 °C. For temperatures above 30 °C, a 25 mm diameter parallel plate with a 1 mm gap was used, while for temperatures below 30 °C, an 8 mm diameter parallel plate with a 2 mm gap was employed.

#### 2.3.4. Frequency Sweep Test

The frequency sweep (FS) test was conducted at temperatures ranging from 15 °C to 75 °C, with increments of 10 °C. This study used the FS test to investigate the viscoelasticity variation in virgin SBS-modified bitumen, ASMB, and three types of rejuvenated SBS-modified bitumen across a wide frequency range. An 8 mm parallel plate was used for temperatures between 15 °C and 35 °C, while a 25 mm parallel plate was employed for temperatures between 45 °C and 75 °C. The shear strain was set at 1%, and the frequency range was 0.1–100 rad/s. The frequency sweep data were processed based on the time–temperature superposition principle, and the Generalized Logistic Sigmoidal model was applied to construct the master curves of the complex modulus and phase angle.

The shift factor α(T) can be calculated using the WLF equation:

(1) $$\mathrm{lg}\alpha \left(T\right)=\frac{-C_{1}\left(T-T_{r}\right)}{C_{2}+T-T_{r}}$$

where *T* and *T_r_* represent the test temperature and the reference temperature (°C), respectively, and *C_1_* and C_2_ are material constants dependent on *T_r_*.

The complex modulus master curve is fitted using the Sigmoidal equation, as shown below:

(2) $$\log\;|G^{*}|=\delta +\frac{\alpha }{1+e^{\beta +\gamma \left({\log f}_{r}\right)}}$$

where ∣*G^∗^*∣ is the complex modulus (Pa), *fr* is the reduced frequency at the reference temperature (rad/s), and *δ*, *α*, *β*, and *γ* are fitting coefficients. The phase angle master curve is calculated using the same approach as the complex modulus master curve [28].

#### 2.3.5. Bending Beam Rheometer Test

This study used the BBR test to investigate the low-temperature cracking resistance of virgin SBS-modified bitumen, ASMB, and three types of rejuvenated SBS-modified bitumen. According to ASTM D6648 [29], the BBR test was conducted to measure the creep stiffness (S) and creep rate (m) of bitumen at temperatures ranging from −24 °C to −6 °C, with increments of 6 °C. In this study, both parameters S and m were combined to calculate a new factor, λ = m/S. A larger λ value indicates a better low-temperature crack resistance of bitumen [30,31].

#### 2.3.6. Fluorescence Microscopy Test

FM was employed in this study to directly observe the polymer distribution within the virgin SBS-modified bitumen, ASMB, and three types of rejuvenated SBS-modified bitumen. For each type of bitumen, more than two samples were randomly taken, and at least six images were captured for each sample.

#### 2.3.7. Fourier Transform Infrared Spectroscopy Test

The ATR accessory was used for sampling with a resolution of 4 cm^−1^, covering a test range of 4000–650 cm^−1^ and performing 4 scans per sample. Solid samples were placed on the ATR crystal and tested directly under pressure from the clamp, while liquid samples did not require clamping. Virgin SBS-modified bitumen, ASMB, and three types of rejuvenated SBS-modified bitumen were tested in triplicate or more to ensure minimal variability between test results.

## 3. Results and Discussion

### 3.1. Multiple Stress Creep and Recovery

**Figure 1:**
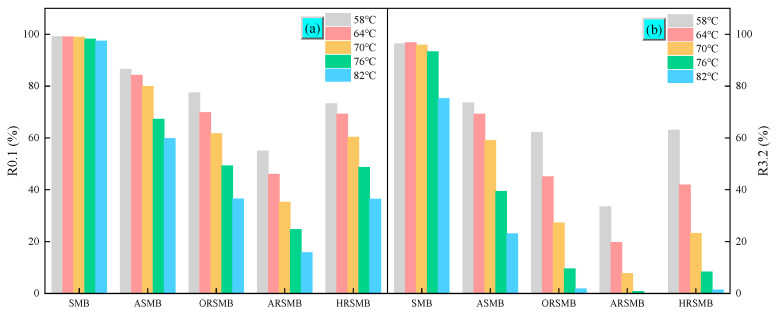
The MSCR test results at different temperatures: (**a**) R0.1, (**b**) R3.2.

The MSCR test simulates the rutting resistance of bitumen under instantaneous loading conditions, reflecting the stress experienced by pavements during service. According to the research conducted by Xing et al. [7], the creep and recovery behavior of bitumen under a stress level of 3.2 KPa is unstable during the initial cycles. When the cycle count is less than eight, the coefficient of variation for each bitumen sample is less than 10%. Therefore, the last eight cycles at 3.2 KPa were selected to calculate R and Jnr. The R of SBS-modified bitumen before and after aging and rejuvenation is shown in Figure 1. To simplify the illustration, SBS-modified bitumen is abbreviated as SMB in the figure, and this abbreviation will be used consistently without further explanation. Virgin SBS-modified bitumen exhibits the highest R at the 0.1 KPa stress level, but R decreases significantly after aging. Rejuvenation with aromatic oil results in a slight further reduction in R, indicating that the elastic properties of SBS-modified bitumen deteriorate after aging and cannot be improved by aromatic oil alone. When the reactive compounds AGE and HDE, combined with BDMA, are added to the aromatic oil, significant differences are observed between the two composite rejuvenators. ARSMB shows a further decrease in R, while HRSMB exhibits no substantial change. This suggests that the reactive compound AGE negatively affects the elastic recovery of bitumen, while HDE has little impact on this property.

**Figure 2:**
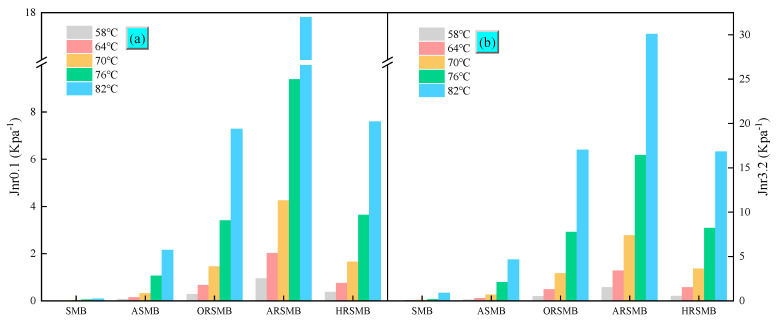
The MSCR test results at different temperatures: (**a**) J_nr_0.1, (**b**) J_nr_3.2.

The Jnr value is commonly used to characterize the high-temperature rutting resistance of bitumen, with smaller Jnr values indicating stronger rutting resistance. Among these, the Jnr data measured at a stress level of 3.2 KPa provides a more accurate representation of rutting resistance under actual road loading conditions, as shown in Figure 2. Virgin SBS-modified bitumen exhibits the lowest Jnr, indicating the best rutting resistance. After aging, Jnr increases significantly, indicating a substantial decline in rutting resistance due to the degradation of SBS, which weakens bitumen’s elastic properties. Rejuvenation with aromatic oil adjusts the composition of aged bitumen and softens it; however, the recovery of elastic properties remains insufficient, further compromising the high-temperature rutting resistance. When the reactive compound AGE and the catalyst BDMA are added, the rutting resistance of bitumen continues to deteriorate. Interestingly, the addition of HDE and BDMA results in no significant further decline in rutting resistance compared to ARSMB. This indicates that the reactive compound HDE does not noticeably harm the high-temperature rutting resistance of bitumen.

### 3.2. Temperature Sweep

**Figure 3:**
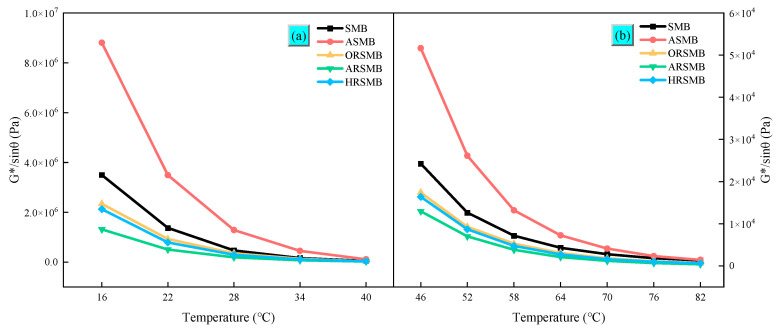
The fatigue factor results for various types of bitumen: (**a**) 16–40 °C; (**b**) 46–82 °C.

The TS test effectively evaluates the rheological properties of bitumen at varying temperatures [32]. This study recorded the complex modulus (G^∗^) and phase angle (θ) from the temperature sweep results and analyzed the fatigue resistance of bitumen at medium-to-high temperatures using the fatigue factor (G^∗^sinθ). The fatigue factor indicates the fatigue resistance of bitumen, with smaller values corresponding to better fatigue performance. As shown in Figure 3, the fatigue factor decreases significantly with increasing temperature. At higher temperatures, bitumen softens and exhibits stronger viscous properties, which in turn enhance the fatigue resistance of the pavement. A longitudinal comparison reveals that the fatigue factor of ORSMB is not only lower than that of ASMB but also lower than that of virgin SBS-modified bitumen. This indicates that aromatic oil significantly improves the flexibility and fatigue resistance of bitumen. Furthermore, both ORSMB and HRSMB exhibit enhanced fatigue resistance compared to virgin SBS-modified bitumen. A comparison reveals that the composite rejuvenator consisting of AGE, BDMA, and aromatic oil (AGE-AO) significantly enhances the fatigue resistance of bitumen compared to the composite rejuvenator containing HDE, BDMA, and aromatic oil (HDE-AO).

To further evaluate the rejuvenation effects of aromatic oil, AGE-AO, and HDE-AO, this study defined a new fatigue rejuvenation variation rate (δ) for subsequent analysis. The calculation formula is as follows:

(3) $$\delta =\left|\frac{{\left(G^{*}sin\theta \right)}_{rejuvenated}-{\left(G^{*}sin\theta \right)}_{age}}{{\left(G^{*}sin\theta \right)}_{age}}\right|$$

where *δ* represents the variation rate of the fatigue factor, *(G^∗^sinθ)_rejuvenated_* denotes the fatigue factor of rejuvenated bitumen, and *(G^∗^sinθ)_aged_* denotes the fatigue factor of aged bitumen.

**Figure 4:**
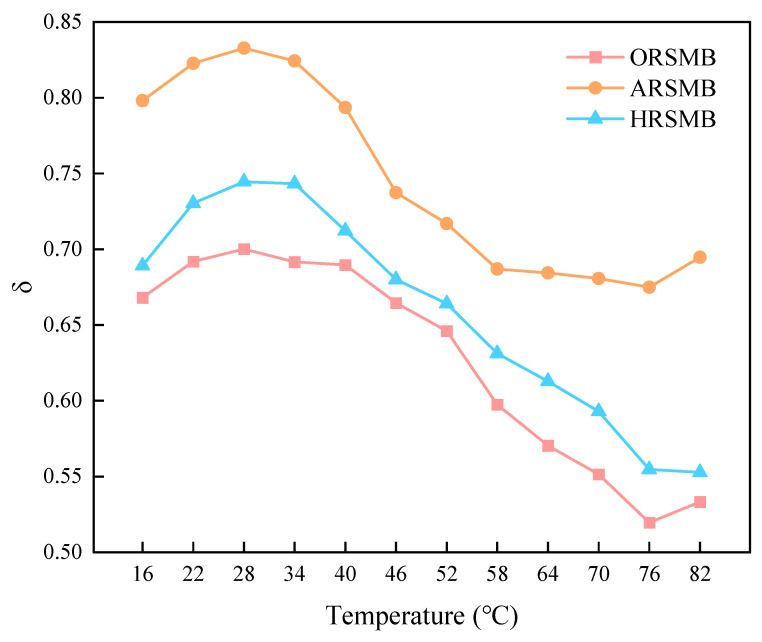
The variation rates of the fatigue factor for different types of bitumen.

Based on the formula, the fatigue rejuvenation variation rates of the three rejuvenators for ASMB were calculated within the temperature range of iI16 °C to 82 °C. As shown in Figure 4, ARSMB and HRSMB exhibit higher rejuvenation effects on bitumen compared to ORSMB. This indicates that AGE and HDE significantly influence the rheological properties of ASMB, particularly in enhancing its flexibility. AGE and HDE further soften the ASMB beyond the effects of aromatic oil, substantially improving ASMB’s fatigue resistance. Additionally, the influence of AGE and HDE becomes more pronounced as the temperature decreases, corresponding to improved fatigue resistance. Overall, at medium and high temperatures, the rejuvenation effects of AGE on aged bitumen are considerably greater than those of HDE, a result consistent with the findings from the MSCR test discussed earlier. Therefore, further investigation is needed to explore the differences in the rejuvenation mechanisms of AGE and HDE.

### 3.3. Frequency Sweep

**Figure 5:**
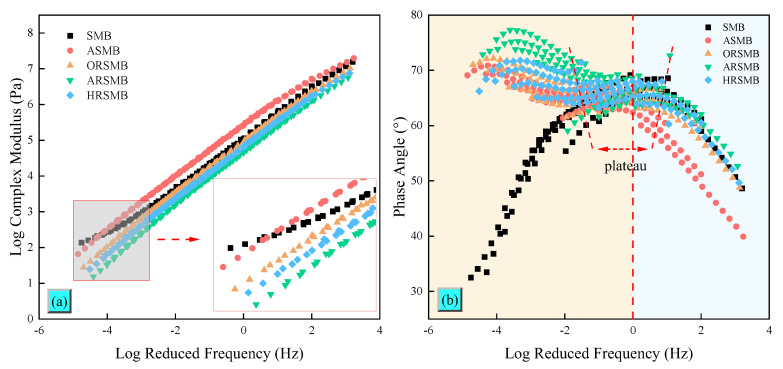
Master curves of the complex modulus and phase angle for different types of bitumen: (**a**) complex modulus, (**b**) phase angle.

The FS test reveals how the properties of bitumen vary under different frequencies, reflecting the rheological behavior in medium and high-temperature environments. Figure 5a,b present the master curves of the complex modulus (G^∗^) and phase angle (θ) for SBS-modified bitumen before and after aging and rejuvenation. As shown in Figure 5a, significant differences in the complex modulus are observed between SBS-modified bitumen before and after aging. In the high-frequency region, the trends for all bitumen types are similar and gradually converge. Among them, the master curve of ASMB consistently remains at the highest position. After adding aromatic oil, the complex modulus of ORSMB decreases sharply. With the addition of reactive compounds AGE and HDE, the complex modulus master curve declines further, with ARSMB exhibiting a lower complex modulus than HRSMB, indicating that AGE has a more pronounced softening effect on bitumen. In the low-frequency region, the arrangement of the complex modulus master curves is similar to that in the high-frequency region. However, it is evident that the slope of the master curve for virgin SBS-modified bitumen decreases with decreasing frequency. This phenomenon is closely related to the presence of SBS, which imparts diverse property characteristics to bitumen. SBS absorbs light components from bitumen, swells, and forms a cross-linked network structure. During the FS test, this structure inhibits the continuous decline in the complex modulus caused by decreasing frequency. At this stage, the polybutadiene structure within the SBS remains active, preventing bitumen softening. As aging progresses, the polybutadiene double bonds in SBS break, and the cross-linked network structure is destroyed, which is one of the primary reasons for the property degradation of aged bitumen. Unfortunately, the added reactive compounds fail to restore this characteristic. Instead, the addition of AGE and HDE further softens bitumen, with AGE exhibiting a more significant softening effect.

The phase angle master curve reflects the viscoelastic property variations in SBS-modified bitumen before and after rejuvenation. As shown in Figure 5b, in the high-frequency region, the phase angle master curves of the bitumen samples before and after rejuvenation decrease with increasing frequency. Among them, the phase angle of ASMB remains consistently lower than that of the other bitumen samples. This is attributed to the higher stiffness and poorer ductility of aged bitumen under high-frequency conditions, resulting in a lower phase angle. After adding aromatic oil, the phase angle master curve shifts upward, indicating an increase in viscosity. The addition of reactive compounds AGE and HDE further increases the phase angle, bringing the phase angle master curves of the two reactive rejuvenated bitumen samples closer to that of virgin SBS-modified bitumen. Interestingly, the phase angle master curve of virgin SBS-modified bitumen in the low-frequency region exhibits a distinct pattern compared to aged bitumen. Specifically, the phase angle decreases with decreasing frequency, which is due to the active state of the polybutadiene segments in SBS. These segments inhibit the increase in viscosity typically caused by decreasing frequency, allowing SBS-modified bitumen to maintain high stability even at elevated temperatures. This unique behavior also accounts for the flat characteristic zone of SBS-modified bitumen in the medium-frequency range. After aging, the cross-linked network structure of SBS-modified bitumen is disrupted, causing the phase angle master curve to exhibit an increasing trend in the low-frequency region. With the addition of rejuvenators, the phase angle master curves of ORSMB, ARSMB, and HRSMB all increase in the low-frequency region. Among these, ARSMB exhibits a noticeably higher phase angle compared to the other two rejuvenated bitumen samples, indicating that AGE has a stronger softening effect on bitumen than HDE.

### 3.4. Ductility and Bending Beam Rheometer Test

#### 3.4.1. Ductility

**Figure 6:**
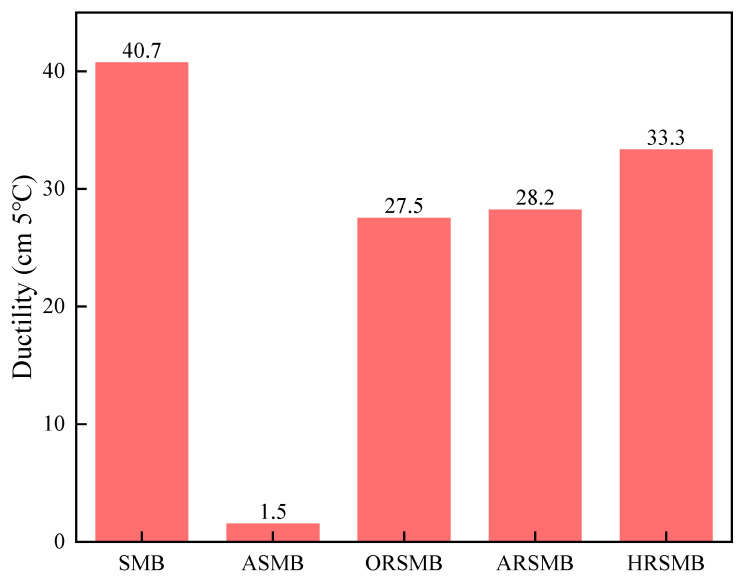
Bar chart of the ductility for different types of bitumen.

The ductility of bitumen refers to its ability to undergo plastic deformation under external force. Higher ductility indicates better plasticity and effectively reflects bitumen’s extensibility at low temperatures. Figure 6 illustrates the ductility variations in SBS-modified bitumen before and after aging and rejuvenation.

Virgin SBS-modified bitumen exhibits a ductility exceeding 40 cm, indicating exceptional low-temperature extensibility. This is primarily attributed to the SBS modifier, which imparts high toughness and flexibility to bitumen under low-temperature conditions. However, aging leads to the degradation of the SBS modifier and the volatilization of light components, significantly reducing the adaptability of bitumen and causing a marked decline in ductility. Upon adding the three rejuvenators, the low-temperature ductility of bitumen improves significantly. The ductility of ORSMB recovers to 27.5 cm, suggesting that aromatic oil can replenish the lost light oil fractions and partially restore bitumen’s low-temperature extensibility. Nevertheless, the SBS modifier remains in a degraded state, resulting in a ductility value still lower than that of virgin SBS-modified bitumen. In contrast, the use of composite rejuvenators containing AGE-AO and HDE-AO further enhances the ductility of ARSMB and HRSMB. Notably, HDE demonstrates a more pronounced improvement in ductility. Previous studies revealed that AGE exhibits a significant softening effect on bitumen at high temperatures, whereas the ductility test indicates that HDE exerts a stronger softening effect at low temperatures. Compared to AGE, HDE not only ensures bitumen’s stability at high temperatures, preventing rapid softening with rising temperatures, but also maintains excellent extensibility at low temperatures—similar to the effects of the SBS modifier. Therefore, it is reasonable to infer that the addition of HDE either produces physical properties akin to SBS or enables the repair of the degraded SBS modifier structure.

#### 3.4.2. Bending Beam Rheometer Test

**Figure 7:**
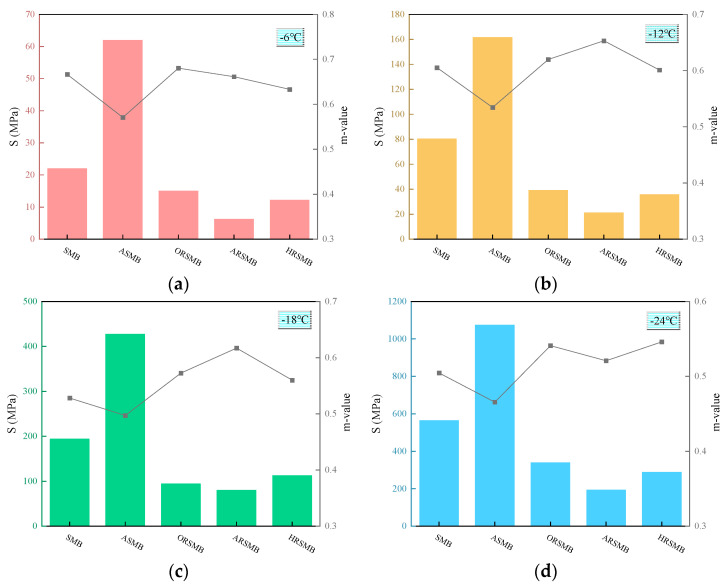
BBR test results of various types of bitumen: (**a**) −6 °C; (**b**) −12 °C; (**c**) −18 °C; (**d**) −24 °C.

The BBR test characterizes the low-temperature crack resistance of bitumen using the creep stiffness modulus (S) and creep rate (m) [33]. S reflects the resistance of bitumen to sustained loading, while m indicates the rate of stiffness change under loading. Figure 7a–d present the variations in S and m for virgin SBS-modified bitumen, aged bitumen, and rejuvenated bitumen under temperatures ranging from −6 °C to −24 °C. The bar charts depict the changes in S, while the line graphs represent the variations in m.

As the temperature decreases from −6 °C to −24 °C, the S values for all types of bitumen increase significantly. Among them, ASMB consistently exhibits the highest S values, whereas ARSMB maintains the lowest S values throughout the temperature range. At −18 °C, the S value of ASMB surpasses 300 MPa, while the S values of virgin SBS-modified bitumen and the three types of rejuvenated bitumen remain below 300 MPa. Overall, bitumen containing the composite rejuvenator of AGE-AO demonstrates the best low-temperature performance.

On the other hand, the m values for all five bitumen types show good stability across the temperature range, likely due to the excellent low-temperature creep properties of the selected 90# bitumen. The trend of m values indicates a gradual decrease as the temperature drops. At −18 °C, ARSMB has the highest m value, while ASMB exhibits the lowest. This further confirms that the composite rejuvenator of AGE-AO provides superior low-temperature rejuvenation performance.

**Figure 8:**
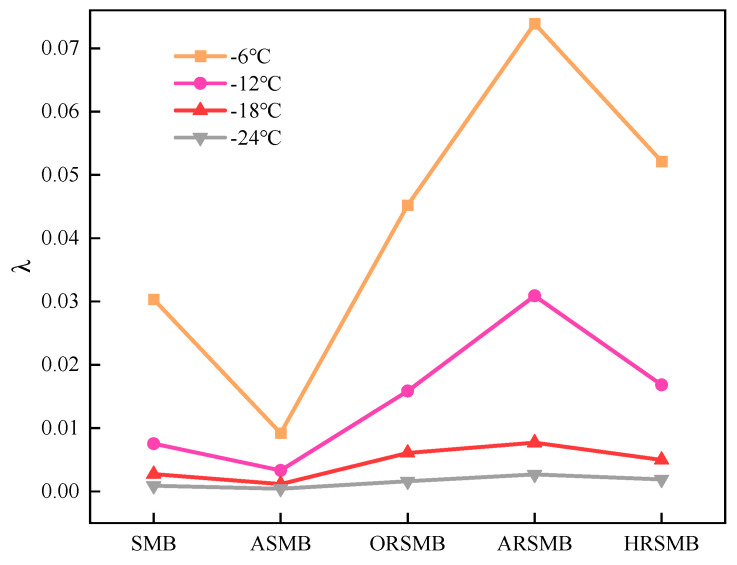
λ values of different types of bitumen.

Based on the study by Liu et al. [30], a new factor, λ = m/S, was calculated by combining the evaluation indicators S and m. The λ factor provides a further assessment of the low-temperature crack resistance of bitumen, with larger λ values indicating stronger resistance to cracking at low temperatures. As shown in Figure 8, the differences in λ values among the various types of bitumen become more pronounced as the temperature increases. At all temperatures, ASMB exhibits the smallest λ values, indicating the poorest low-temperature crack resistance. In contrast, ARSMB consistently has the highest λ values, demonstrating the strongest low-temperature crack resistance. ORSMB maintains good low-temperature crack resistance across the temperature range but falls short compared to ARSMB. HRSMB’s λ values are similar to those of ORSMB, suggesting that the reactive compound HDE has a relatively minor impact on low-temperature crack resistance. Overall, the composite rejuvenator of AGE and aromatic oil significantly enhances the low-temperature crack resistance of bitumen, particularly under low-temperature conditions.

#### 3.4.3. Relationship Between Ductility and Bending Beam Rheometer Test

Ductility and BBR test results are critical indicators for evaluating the low-temperature properties of bitumen, with each method focusing on different performance characteristics and perspectives [34]. The ductility test assesses the extensibility and toughness of bitumen at low temperatures, while the BBR test measures the deflection of a bitumen beam under a constant load, characterizing resistance to deformation and strain rate. Comparatively, the ductility test provides an overall measure of low-temperature extensibility but fails to capture strain and relaxation behavior under complex stress conditions. In contrast, the BBR test focuses on mechanical behaviors, including elastic deformation and strain rate, offering complementary insights. Combining both methods is essential for a comprehensive understanding of bitumen’s low-temperature performance. In this study, virgin SBS-modified bitumen exhibits excellent performance in both ductility and λ values, demonstrating an optimal combination of low-temperature extensibility and elastic recovery capacity. However, aging significantly reduces both the ductility and low-temperature crack resistance of bitumen. After rejuvenation with aromatic oil, ductility and low-temperature crack resistance improve, but the overall extensibility remains inferior to virgin SBS-modified bitumen, largely due to the unique contributions of SBS modifiers.

The introduction of two reactive rejuvenators markedly enhances the low-temperature properties of aged bitumen. Both ductility and λ values exceed those of virgin SBS-modified bitumen, indicating superior rejuvenation effects compared to aromatic oil alone. Interestingly, the two reactive compounds exhibit different strengths: AGE is more effective in increasing λ values, while HDE significantly enhances ductility. This suggests that AGE and HDE target different aspects of low-temperature property improvement in bitumen.

### 3.5. Fluorescence Microscopy

**Figure 9:**
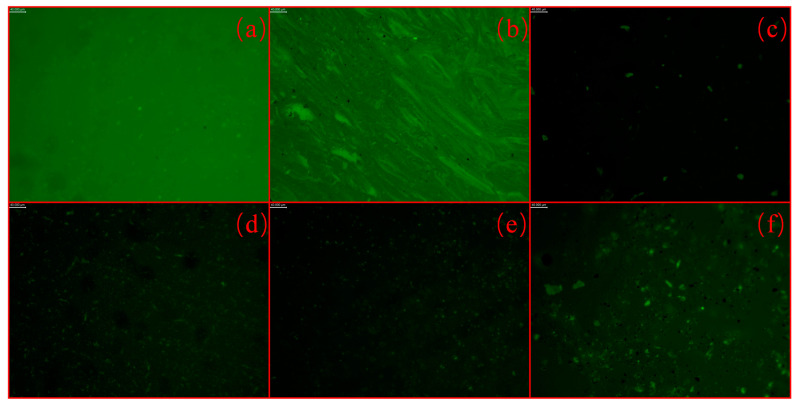
Fluorescence images of different types of bitumen: (**a**) base bitumen; (**b**) SMB; (**c**) ASMB; (**d**) ORSMB; (**e**) ARSMB; (**f**) HRSMB.

FM provides a valuable method for analyzing the microstructure, polymer distribution, and aging characteristics of SBS-modified bitumen [35,36]. Figure 9a–f present fluorescence images of base bitumen, SBS-modified bitumen, ASMB, ORSMB, ARSMB, and HRSMB, respectively.

Figure 9a shows the fluorescence image of 90# base bitumen without the SBS modifier. The green background is a result of instrument-adjusted brightness, a normal phenomenon for such samples. In SBS-modified bitumen, the SBS modifier generates a fluorescence response, as illustrated in Figure 9b. In contrast, Figure 9c depicts the fluorescence image of ASMB, where polymer degradation has occurred. The background color is significantly darker, and only sparse fluorescence points remain visible [37]. After the addition of aromatic oil, the fluorescence image of rejuvenated bitumen, shown in Figure 9d, reveals finer fluorescence distribution, likely due to the diluting effect of the aromatic oil, which promotes a more uniform dispersion of SBS. Further, Figure 9e,f display fluorescence images of bitumen rejuvenated with AGE–aromatic oil and HDE–aromatic oil, respectively. Compared to Figure 9d, the fluorescence points in Figure 9e do not exhibit significant changes. However, in Figure 9f, the fluorescence points are noticeably larger and more vivid in color.

These observations suggest that the reactive compound HDE might contribute to repairing degraded SBS, partially restoring the fluorescence characteristics. In contrast, AGE appears to have limited efficacy in improving SBS degradation, as indicated by the relatively unchanged fluorescence patterns.

### 3.6. Fourier Transform Infrared Spectroscopy

**Figure 10:**
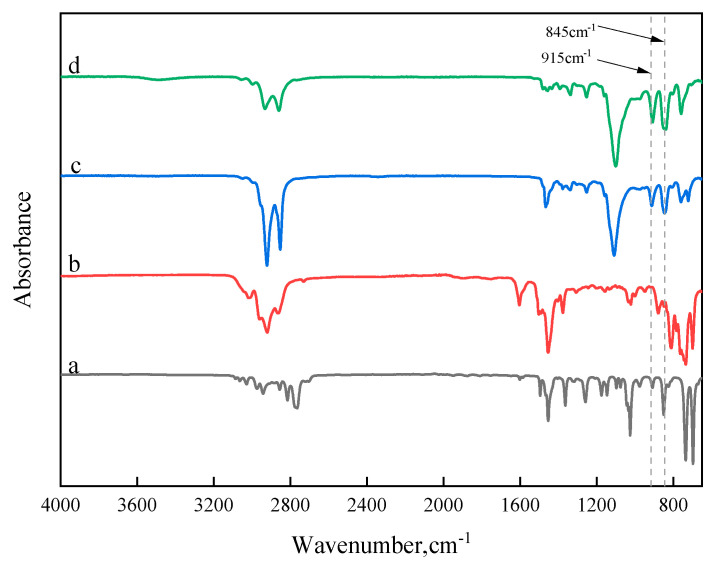
Infrared spectra of individual additives: (a) BDMA; (b) aromatic oil; (c) AGE; (d) HDE.

FTIR is an effective tool for analyzing the functional group structural changes in bitumen molecules [38]. By examining changes in infrared spectral peaks, it is possible to infer the degree of bitumen aging and assess whether chemical reactions occur between bitumen and rejuvenators. Figure 10a–d present the FTIR spectra of the additives used in this study, including BDMA, aromatic oil, AGE, and HDE. BDMA, as a catalyst, does not react with bitumen or other additives, and its low dosage of only 0.1% makes it a minor focus of this study. The characteristic peaks of epoxy groups are typically observed in the 800–950 cm^−1^ range, with particular attention to the absorption peaks at 845 cm^−1^ and 915 cm^−1^ for AGE and HDE. Compared to aromatic oil, the reactive compounds AGE and HDE exhibit distinct vibrational absorption peaks at these positions. By comparing the peak intensities, it is evident that HDE exhibits stronger peak intensities than AGE, likely due to HDE’s diepoxy structure, whereas AGE contains only a single epoxy group.

**Figure 11:**
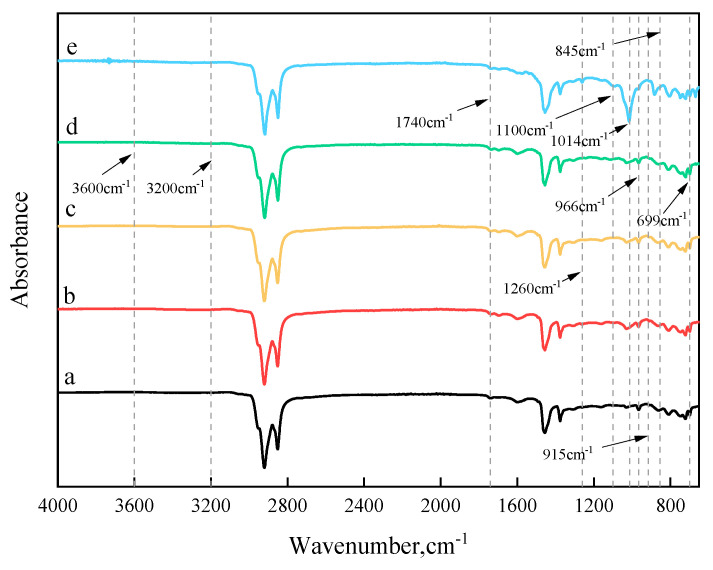
Infrared spectra of various types of bitumen: (a) SMB; (b) ASMB; (c) ORSMB; (d) ARSMB; (e) HRSMB.

By comparing the functional group changes in SBS-modified bitumen before and after aging and rejuvenation, the rejuvenation mechanism of reactive compounds during the bitumen rejuvenation process can be explored. The overall changes in infrared spectral peaks of various bitumen samples are illustrated in Figure 11. HRSMB exhibits a unique peak at 1014 cm^−1^ that is absent in the other bitumen samples, corresponding to ester groups (-COOR). The stretching vibration of the C-O bond in ester groups typically appears near 1000 cm^−1^. Additionally, HRSMB displays a distinctive peak change around 1260 cm^−1^, which is also likely attributed to ester groups (C-O-C). In contrast, no ester-related functional groups are observed in ARSMB.

**Figure 12:**
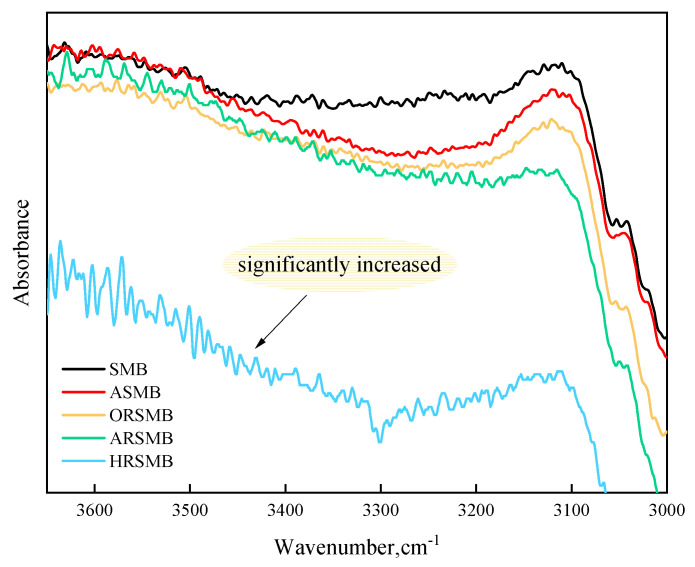
Enlarged view of a specific region of the infrared spectrum.

Furthermore, Figure 12 presents a direct comparison of peak intensity differences within the 3200–3600 cm^−1^ range without vertical axis adjustment, where hydroxyl groups predominantly appear. The stretching vibrations of hydroxyl groups typically occur between 3200 and 3600 cm^−1^ and exhibit a broad peak due to hydrogen bonding effects. HRSMB shows the strongest peak signal in this region, distinctly different from the other bitumen samples. This phenomenon arises because epoxy groups undergo ring-opening addition reactions with hydroxyl groups. In such reactions, hydroxyl groups act as nucleophiles, attacking one carbon atom of the epoxy group, leading to ring-opening and the formation of diols. Similarly, epoxy groups can react with carboxyl groups in a ring-opening process. In this case, the carbonyl oxygen (C=O) of the carboxyl group acts as a nucleophile, attacking the epoxy group, resulting in ring cleavage and the formation of an ester group and a hydroxyl group. These combined effects explain the intensified peaks observed at 3200–3600 cm^−1^ in HRSMB, which fundamentally indicate an increased hydroxyl concentration.

In summary, it is evident that HDE, as a reactive compound, can chemically react with the hydroxyl and carboxyl groups at the degraded SBS chain ends, whereas AGE does not exhibit this capability. The dual epoxy groups in HDE undergo ring-opening reactions under the action of the catalyst, allowing them to separately react with hydroxyl or carboxyl groups in SBS, effectively reconnecting the broken SBS structure. Conversely, AGE contains only a single epoxy group. While the catalyst facilitates its ring-opening reaction, AGE can only react with either the hydroxyl or carboxyl group of the broken SBS chain, making it incapable of reconnecting the two ends of the polybutadiene chain. Consequently, the rejuvenation mechanism of AGE for aged bitumen is more akin to that of light oil fractions.

### 3.7. Comprehensive Analysis

Through comprehensive macro- and micro-scale experiments, this study meticulously investigated the rejuvenation effects of reactive compounds AGE and HDE on SBS-modified bitumen. By analyzing both macro- and micro-properties, this study explored the mechanisms underlying the rejuvenation effects of the two composite rejuvenators. The experimental results demonstrated that HDE, with its dual-epoxy structure, significantly improved the properties of ASMB, outperforming AGE (mono-epoxy structure) due to its superior chemical reactivity. HDE facilitated the reconnection of broken molecular chains in degraded SBS by reacting with terminal hydroxyl and carboxyl groups, restoring the elasticity of SBS while significantly enhancing the ductility and crack resistance of rejuvenated bitumen under low-temperature conditions.

In contrast, AGE, limited by its mono-epoxy structure, primarily improved bitumen properties through physical softening by reducing viscosity and enhancing low-temperature performance without repairing molecular chain structures. Consequently, AGE exhibited excellent physical rejuvenation effects, characterized by notable softening and enhanced low-temperature crack resistance. However, high-temperature performance tests revealed that, despite effectively repairing degraded SBS, HDE-rejuvenated bitumen did not show proportional improvement in high-temperature properties. This was attributed to the flexible chain structure of HDE, which introduced a softening effect. The combined effects of HDE-induced softening and SBS repair-induced stiffening stabilized the high-temperature rutting resistance but slightly reduced it overall. Conversely, AGE’s more pronounced softening effect, albeit without SBS chain repair, further decreased the high-temperature rutting resistance.

In summary, HDE, containing di-epoxy groups, demonstrated superior chemical rejuvenation effects, making the material highly suitable for repairing aged SBS-modified bitumen. In contrast, AGE, though incapable of restoring SBS molecular structures, exhibited strong physical softening characteristics, making it better suited for rejuvenating base bitumen, with promising application potential.

## 4. Conclusions

This study comprehensively evaluated the high-, medium-, and low-temperature properties of SBS-modified bitumen before and after aging and rejuvenation from both macroscopic and microscopic perspectives. It focused on analyzing the rejuvenation mechanisms of AGE-BDMA–aromatic oil composite rejuvenators and HDE-BDMA–aromatic oil composite rejuvenators on aged bitumen. Based on the findings, the main conclusions are as follows:

(1) From the perspective of the three composite rejuvenators, ASMB rejuvenated with the two reactive rejuvenators demonstrates significantly better fatigue resistance and low-temperature properties compared to AORSMB. However, the high-temperature rutting resistance is inferior to that of AORSMB.
(2) Regarding the individual effects of the reactive compounds AGE and HDE, at high temperatures, AGE shows a strong softening effect, significantly impairing the high-temperature rutting resistance of ASMB. In contrast, HDE causes a much smaller reduction in ASMB’s high-temperature rutting resistance. At medium temperatures, both AGE and HDE moderately enhance the fatigue resistance of ASMB. At low temperatures, both significantly improve the low-temperature properties of ASMB.
(3) Microscopic analysis reveals that the mono-epoxy structure of AGE cannot chemically react with degraded SBS. AGE’s rejuvenation mechanism for ASMB relies on physical softening, improving the low-temperature properties while compromising the high-temperature rutting resistance. On the other hand, the di-epoxy structure of HDE, under the catalytic action of BDMA, chemically reacts with degraded SBS, reconnecting broken molecular chains and thereby enhancing ASMB’s low-temperature properties.
(4) The flexible chain structure of HDE imparts a certain softening effect on ASMB, while HDE’s ability to reconnect and repair degraded SBS imparts rigidity. These effects jointly result in minimal changes to the high-temperature rutting resistance of rejuvenated bitumen.
(5) Due to the differing rejuvenation mechanisms of AGE and HDE, composite rejuvenators containing AGE primarily enhance the low-temperature crack resistance of bitumen through physical rejuvenation, making them more suitable for rejuvenating base bitumen. Conversely, composite rejuvenators containing HDE improve the low-temperature ductility of bitumen by chemically repairing degraded SBS, making them more appropriate for rejuvenating SBS-modified bitumen.

## Figures and Tables

**Table 1:** Basic properties of base bitumen and corresponding SBS-modified bitumen.

Type	Base Bitumen	SBS-Modified Bitumen
Penetration (25 °C)/0.1 mm	83	71
Softening Point (℃)	46	81
Ductility (5 °C, cm)	/	40.7
Ductility (15 °C, cm)	>100	/
Flash Point	/	>230

**Table 2:** Basic properties of aromatic oil.

Viscosity @150 °C (mm^2^/s)	Flash Point (℃)	Deterioration Rate (%)	Density @20 °C (g/mL)	Distillation Range@2% (℃)
1.20	174	2.5	1.012	310

**Table 3:** Basic properties of AGE, HDE, and BDMA.

Item	AGE	HDE	BDMA
Molecular formula	C_48_H_96_O_6_	C_12_H_22_O_4_	C_9_H_13_N
Molecular weight(g/mol)	769.27	230.30	135.206
Flash point (°F)	>230	248	129.92
Density at 25 °C	0.89 (g/mL)	1.076 (g/mL)	0.9 (g/mL)

## Data Availability

The original contributions presented in this study are included in the article. Further inquiries can be directed to the corresponding authors.
